# Elevated expression of IL-17RB and ST2 on myeloid dendritic cells is associated with a Th2-skewed eosinophilic inflammation in nasal polyps

**DOI:** 10.1186/s13601-018-0237-4

**Published:** 2018-11-29

**Authors:** Rui Zheng, Dan Wang, Kai Wang, Wen-Xiang Gao, Qin-Tai Yang, Li-Jie Jiang, Min Zhou, Yu-Jie Cao, Jianbo Shi, Yueqi Sun

**Affiliations:** 1 Otorhinolaryngology Hospital, The First Affiliated Hospital, Sun Yat-sen University, 58 Zhongshan Road II, Guangzhou, 510080 Guangdong China; 2 Guangzhou key Laboratory of Otorhinolarygology, Guangzhou, 510080 China; 3 Department of Otorhinolaryngology-Head and Neck Surgery, First People’s Hospital of Foshan, Foshan, 528000 China; 4 Department of Otorhinolaryngology-Head and Neck Surgery, The Third Affiliated Hospital, Sun Yat-sen University, Guangzhou, 510630 China

**Keywords:** Nasal polyps, Dendritic cells, Interleukin-25, Interleukin-33, Thymic stromal lymphopoietin

## Abstract

**Background:**

Interleukin(IL)-25, IL-33, and thymic stromal lymphopoietin (TSLP) underlie the crosstalk between epithelial cells and dendritic cells (DCs) during the development of Th2 responses. This study aimed to measure the expressions of IL-17RB, ST2 and TSLPR, receptor of IL-25, IL-33, and TSLP respectively, on myeloid DCs in nasal polyps (NP) and evaluate their association with local Th2 inflammation and disease severity in patients with NP.

**Methods:**

Samples were collected from 30 NP patients and 16 control subjects recruited prospectively. The mRNA expression of cytokines, including TSLP, IL-25 and IL-33, as well as interferon (IFN)-γ, IL-4, IL-5, IL-13 and IL-17A in NP and control tissues was examined by qualitative polymerase chain reaction (qPCR). The expression of IL-17RB, ST2 and TSLPR as well as other surface markers on myeloid DCs (mDCs) was examined by flow cytometry.

**Results:**

Increased numbers of total and activated mDCs were found in NP patients. mDCs demonstrated significantly higher expression of IL-17RB, ST2 and TSLPR than those in control tissues. The activated mDCs exhibited up-regulations of OX40L and ICOSL, but down-regulation of PDL1 in NP. Moreover, the IL-17RB, ST2 and TSLPR levels on mDCs were positively correlated with IL-25, IL-33 and TSLP mRNA levels, respectively, in NP. Furthermore, IL-17RB and ST2 expressions on mDCs were correlated with the IL-5 mRNA level as well as eosinophil number in NP. Importantly, the IL-17RB expression on mDCs and the OX40L expression on activated mDCs in NP were positively correlated with CT score and total nasal symptom score.

**Conclusions:**

Increased expressions of IL-17RB and ST2 on mDCs are associated with enhanced local Th2 inflammation in NP, suggesting that mDCs might play a role in IL-25- and IL-33-induced type 2 responses and eosinophilic inflammation in NP.

**Electronic supplementary material:**

The online version of this article (10.1186/s13601-018-0237-4) contains supplementary material, which is available to authorized users.

## Introduction

Chronic rhinosinusitis (CRS), affecting 14% of adults in the United States and 8% in China [1, 2], is a chronic mucosal inflammation mediated by innate and adaptive immune cells and mediators in nasal sinus. CRS is typically classified into two types of clinical phenotypes: chronic rhinosinusitis without nasal polyps (CRSsNP) and chronic rhinosinusitis with nasal polyps (CRSwNP) [1]. Although emerging evidences have indicated that different regions of the world might have different endotypes of CRS [3–6], a T helper (Th) 2-predominant eosinophilic endotype in NP has been well documented in the western world [3, 7–9], and notably has increased over past 10 years in oriental countries, such as Thailand [10] and Korea [11]. However, the cellular and molecular mechanisms driving the Th2-predominant immune response in NP remain unclear.

With potent antigen presenting capacity, dendritic cells (DCs) are a heterogeneous population of cells, consisting of multiple subtypes. It is clear now that different DC subsets perform different tasks. For instance, some subsets of DCs are better at cross-presentation of antigen to CD8 T cells on major histocompatibility complex (MHC)- I molecules and others better at presenting endocytosed antigen to CD4 T cells on MHC-II molecules [12, 13]. On the other hand, DCs can be divided into functional subsets according to their polarizing function on naïve T cells, such as a Th1-skewing subset and a Th2- skewing subset, which usually are both differentiated from myeloid DCs (mDCs) [14]. In NP, it has been reported that mDCs are increased in NP tissues [15, 16], and two distinct DC subsets, OX40L/PDL1^+^ DCs with Th2-cell-priming ability and low OX40L/PDL1-expressing DCs with Th1/17-cell-inducing ability, are associated with forming eosinophilic and non-eosinophilic endotype of NP respectively [17], indicating an important role DCs play in the modulation of T cell response in NP. However, the molecular factors in regulating functional DC subsets to induce Th2 inflammation in NP have not been fully understood.

Thymic stromal lymphopoietin (TSLP), IL-25, and IL-33 are three cytokines predominantly produced by epithelial cells at mucosal surfaces in response to a wide range of environmental stimuli, and their expression during type 2 diseases, including NP, in humans has been widely documented [18–26]. It is now evident that these cytokines play an important role in initiating type 2 immunity in mammals by activating resident mucosal group 2 innate lymphoid cells (ILC2s) to produce Th2-type cytokines (IL-5 and IL-13) and skewing CD4+ T cells toward Th2 differentiation [27]. Furthermore, accumulating evidence show that these cytokines can also activate DCs to induce Th2-type immune responses. TSLP induces DC activation in nasal mucosa and enhances their capacity to initiate Th2 responses [28]. In a mouse model of house dust mite-induced airway inflammation, IL-25 was shown to promote Th2 and Th9 inflammation in lungs by targeting DCs [29]. Upon IL-33 exposure, DCs exhibited increased expression of CD40 and OX40 ligand (OX40L) and became very potent at inducing Th2 responses [30]. In addition, we recently reported that mDCs in peripheral blood mononuclear cells (PBMCs) in atopic subjects expressed higher levels of IL-17RB (IL-25 receptor), ST2 (IL-33 receptor) and TSLPR (TSLP receptor) than those of non-atopic subjects [31]. Of note, DCs generated from peripheral blood monocytes of atopic subjects with GM-CSF and IL-4 in vitro also expressed higher level of IL-17RB and could enhance a Th2-type response, suggesting that DCs expressing IL-17RB might be a Th2-skewing subset [31]. However, whether mDCs in NP have similar receptor expression patterns as those seen in atopic subjects remains unknown.

The aim of this study was to explore the phenotypic characteristics of mDCs, especially the expression of IL-17RB, ST2 and TSLPR, and their potential contribution to the Th2 inflammation and disease severity in NP.

## Materials and methods

### Subjects

The study was approved by the Ethics Committee of the First Affiliated Hospital, Sun Yat-sen University, and conducted with written informed consent from each patient. All subjects were prospectively recruited at the First Affiliated Hospital of Sun Yat-sen University in Guangzhou. The inclusion criteria for patients with NP were diagnosis of NP, which was made based on the European Position Paper on Rhinosinusitis and Nasal Polyps 2012 guidelines [1], and bilateral NP. The inclusion criteria for control subjects were patients undergoing optic nerve decompression for traumatic optic neuropathy without any sinonasal disease or allergic rhinitis. Only adult subjects were recruited in this study. Anyone who had taken oral or nasal corticosteroids or other medications (e.g., antibiotics or antileukotrienes) for 4 weeks before sample collection was excluded. Other exclusion criteria included those who were pregnant or breastfeeding, patients with cystic fibrosis, immune deficiency, sinonasal tumor or any other severe concurrent disorders. Peripheral blood samples were obtained before surgery for flow cytometry analysis of circulating DC subsets; NP tissues from NP patients and uncinate process mucosa from control subjects were obtained during surgery for flow cytometry, quantitative RT-PCR and histology. The computed tomography (CT) score (range: 0–24) was evaluated by preoperative CT scans using the Lund-Mackay CT scoring system [32]. The endoscopic score (ES) of bilateral nasal polyps was evaluated (range: 0–8) by nasal endoscopy as previously described [33]. The total nasal symptom score (TNSS) was calculated (range: 0–12) by adding up the individual nasal scores including nasal congestion, anterior rhinorrhea, postnasal drip, and loss of smell, each evaluated using a scale of 0 = None, 1 = Mild, 2 = Moderate, or 3 = Severe [6] (Additional file 1: Table S5). The atopic status was evaluated by using assays for specific IgE (HOB Biotech Group, Suzhou, China) against the local common inhalant allergens. Specific IgE concentrations above 0.35 IU/mL were considered positive. The percentages of blood eosinophils in total white blood cells were detected by blood routine test, and percentages above 5% were considered elevated. The NP tissues were stained with hematoxylin–eosin (HE) method to calculate the numbers of tissue eosinophils/high power field (HPF) in NP mucosa as well as the percentages of eosinophils in the total infiltrating cells, and percentages above 10% were considered elevated [5]. The diagnosis of asthma and aspirin tolerance was performed by a specialist physician and was established according to the Global Initiative for Asthma 2006 guideline [34].

### Flow cytometry

PBMCs were isolated by means of Ficoll-Hypaque gradient centrifugation method as previously described [35]. Tissue specimens were rinsed with DMEM/F12 medium containing 1% penicillin/streptomycin (Gibco, Carlsbad, California) and 3.4 μg/mL amphotericin B (Dingguo, Beijing, China) to remove residual blood. Then, tissues were finely minced and digested with collagenase type II (2 mg/mL) and Deoxyribonuclease I (0.1 mg/mL) (Sigma-Aldrich, St Louis, Missouri) at 37 °C with stirring for 1 h. The digested fragments were grinded and filtered through a mesh of 40 μm and a single cell suspension was obtained, and then the tissue mononuclear cells (TMCs) were isolated using Ficoll-Hypaque gradient centrifugation method. DC subsets in TMCs and PBMCs were identified by staining with a cocktail of monoclonal antibodies. Specifically, PBMCs and TMCs were incubated at 4 °C for 30 min with florescence-conjugated monoclonal antibodies against CD1c, CD86, IL-17RB, TSLPR, OX40L, PDL1, ICOSL, and polyclonal antibodies against ST2. Fluorescence minus one (FMO) controls were also prepared for each marker. Species and subtype-matched isotype control antibodies were also used in FMOs for each sample. Positive gate for each marker in each sample was determined by using less than 1% of events on FMO samples. The flow cytometry was performed on a Beckman Coulter Gallios, and data were analyzed with Kaluza Analysis 1.3 (Kaluza software, Fullerton, CA, USA). Antibodies used in flow cytometry are listed in Additional file 1: Table S1.

### Quantitative RT-PCR

Total RNA was extracted from tissue samples by using RNAiso Plus reagent and cDNA was reverse transcribed by using PrimeScript™ RT Master Mix kit (both from TaKaRa, Shiga, Japan) following the manufacturer’s instructions. The quantitative PCR of innate and adaptive cytokines (IL-25, IL-33, TSLP, IFN-γ, IL-4, IL-5, IL-13 and IL-17A) was performed by using the FastStart Universal SYBR Green Master kit (Roche, Mannheim, Germany) with appropriate primers. GAPDH was used as an endogenous reference. The sequences of primers are listed in Additional file 1: Table S2. Amplification was carried out on the CFX96™ Real-Time PCR cycler (Bio-Rad, CA, USA) using the cycling conditions as follows: 10 min’ initial denaturation at 95 °C, 40 cycles consisted of 10 s at 95 °C and 30 s at 60 °C. The melting curve was obtained from 60 to 95 °C (0.5 °C/s). Expression of target gene was expressed as fold increase relative to the expression of GAPDH. The mean value of the replicates for each sample was calculated and expressed as cycle threshold (Ct). The amount of gene expression was then calculated as the difference (ΔCt) between the Ct value of target gene and the Ct value of GAPDH. Fold changes in target gene mRNA were determined as 2^−ΔCt^ [36].

### Statistical analysis

Statistical analysis was performed using GraphPad Prism 6 (GraphPad Software, San Diego, CA, USA). The normality of the data was tested by Shapiro–Wilk test. For normally distributed variables, data are presented as mean with standard deviation (SD). For abnormally distributed variables, data are presented as median with interquartile range (IQR). The t test, Mann–Whitney U test or Chi square (χ^2^) test was used to compare differences between groups. The Spearman’s rank correlation coefficient was used to analyze the correlations. A *P* value of less than 0.05 was considered significant.

## Results

### Patient characteristics

Thirty patients with NP and sixteen control subjects were included. The clinical characteristics of the study subjects are presented in Table 1. Age and gender were matched between the two study groups. The percentage of atopy in patients with NP was 23.3%. Fourteen patients (46.7%) were eosinophilic NP based on a previous criterion [5].

**Table 1:** Clinical characteristics of patients with NP and control subjects

Age (years), mean (SD)	Patients (n = 30)		Control (n = 16)		Test	*P* value
	39.67 (12.47)		41.13 (11.40)		0.39^a^	0.69
	Number	%	Number	%		
Gender						
Male	18	60	10	62.5	0.03^b^	0.87
Female	12	40	6	37.5		
Patients with atopy	7	23.3	0	0		
Patients with elevated blood eosinophils	12	40	0	0		
Patients with elevated tissue eosinophils	14	46.7	0	0		
Patients with asthma	6	20	0	0		
Patients with aspirin intolerance	0	0	0	0		
Patients with smoking	1	3.3%	1	6.3%		
TNSS score	7.0 (6.0–8.2)		N/A			
CT score	18.0 (11.7–22.0)		N/A			
Endoscopic score	5.5 (4.0–6.0)		N/A			

For TNSS, CT score and endoscopic score, results are expressed as medians and interquartile ranges

*TNSS* total nasal symptom score, *CT* computed tomography, *N/A* not applicable

^a^t-test

^b^Chi square (χ^2^) test

### Increased mRNA expression profile of innate and adaptive cytokines in NP tissues

**Fig. 1:**
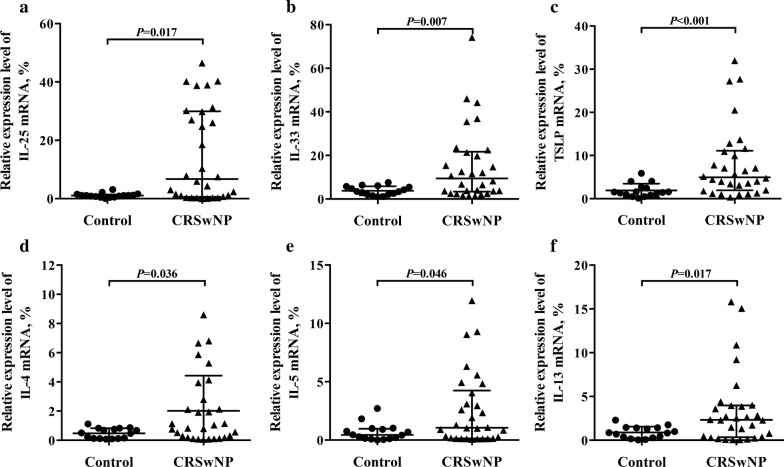
Relative mRNA expression levels of cytokines in NP tissues. Epithelium-derived cytokines IL-25 (**a**), IL-33 (**b**) and TSLP (**c**); Th2 cytokines IL-4 (**d**), IL-5 (**e**) and IL-13 (**f**) were measured by quantitative RT-PCR. In control group, n = 16; in NP group, n = 30. Data are presented as mean with SD or median with IQR

We first compared the mRNA expression profile of innate and adaptive cytokines, including IL-25, IL-33, TSLP, IFN-γ, IL-4, IL-5, IL-13 and IL-17A, between NP and healthy control tissues. Consistent to previous reports [23–25], mRNA levels of IL-25, IL-33, TSLP, IL-4, IL-5 and IL-13 were significantly higher in NP tissues than those in control tissues (Fig. 1a–f). However, no significant differences in IFN-γ and IL-17A mRNA levels were detected between NP and control tissues (data not shown).

### Increased numbers of total and activated myeloid DCs in NP

**Fig. 2:**
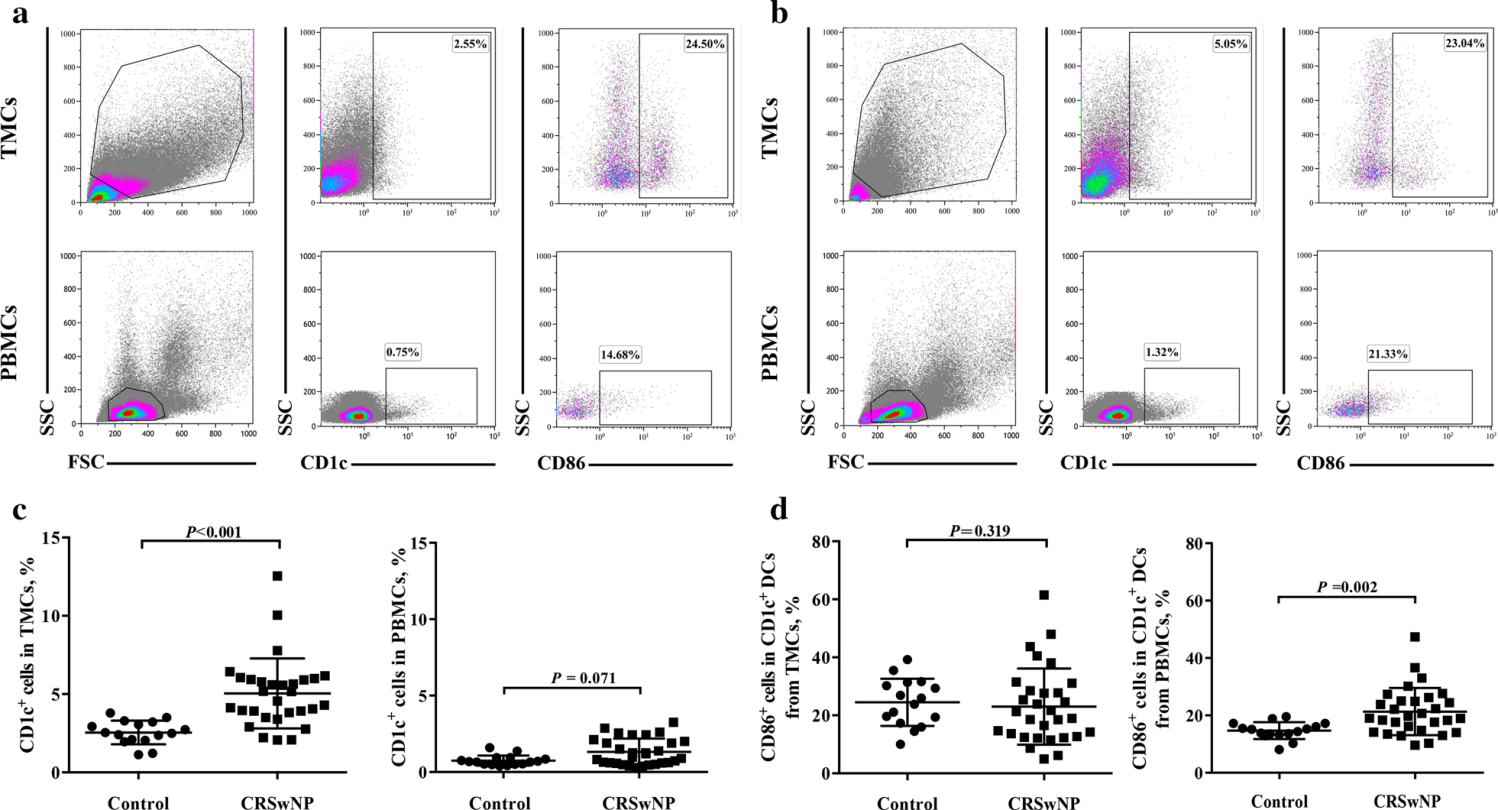
Percentages of total and activated CD1c^+^ DCs in NP tissues and blood. Representative flow-cytometry pseudocolor density plots, in which pink-blue-green–red indicates increasing cell density, showing the percentages of total CD1c^+^ DCs and CD86^+^CD1c^+^ DCs in nasal tissues and blood from healthy controls (n = 16) (**a**) and polyp tissues and blood from NP patients (n = 30) (**b**). Quantification of the percentages of total CD1c^+^ DCs (**c**) and CD86^+^CD1c^+^ DCs (**d**) in NP tissue and blood. Data are presented as mean with SD or median with IQR

Next, we compared the numbers of total and activated myeloid (CD1c^+^) DCs in nasal tissues and blood between NP patients and control subjects. The percentage of the total CD1c^+^ cells was increased in NP tissues, but not in PBMCs, compared with that of control subjects (Fig. 2a–c). By contrast, the percentage of the CD86^+^CD1c^+^ cells was higher in the PBMCs of NP patients, but not in NP tissues, than that of control subjects (Fig. 2a, b, d).

### Increased expressions of IL-17RB, ST2 and TSLPR on CD1c^+^ DCs in NP tissues

**Fig. 3:**
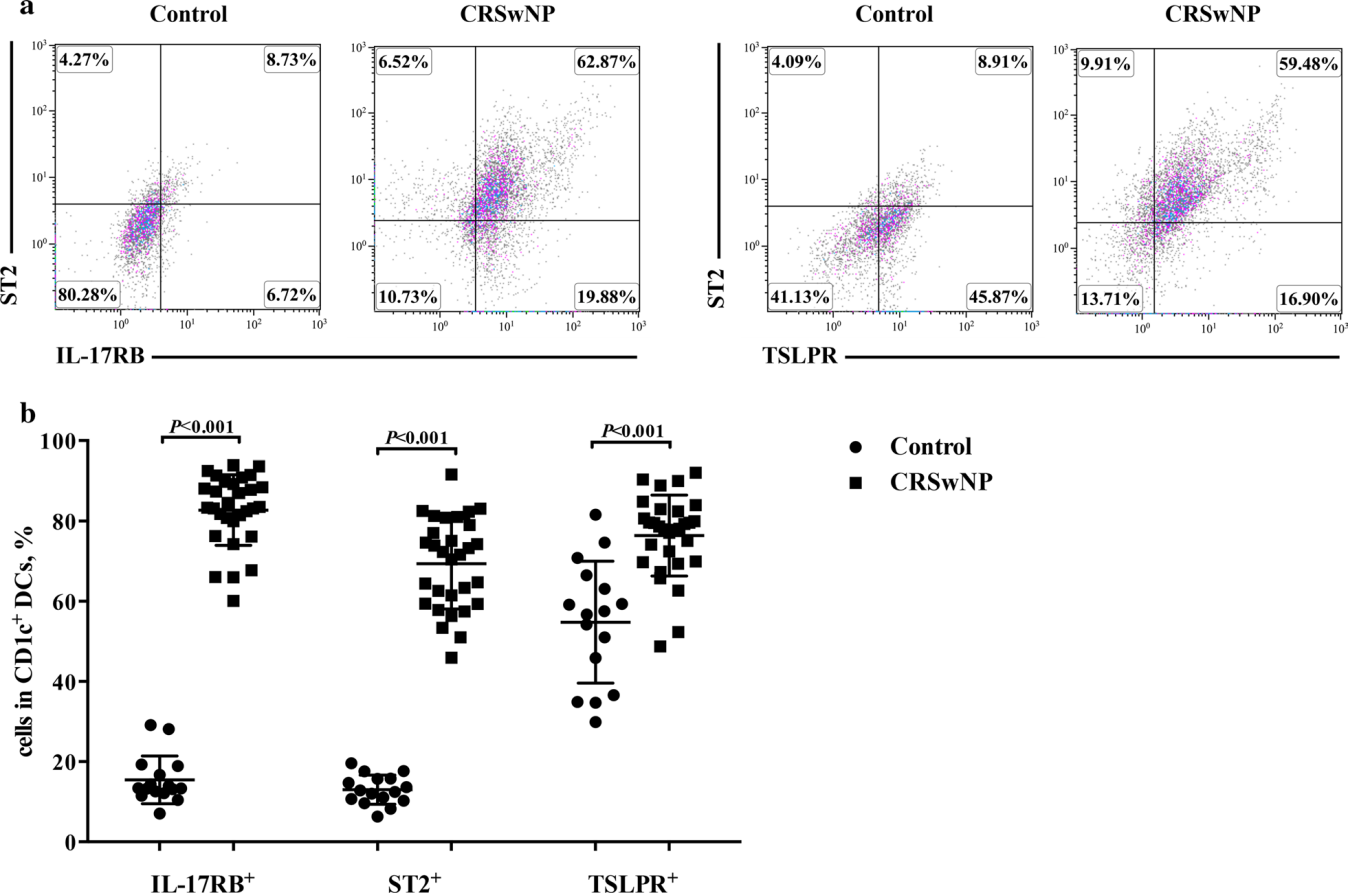
Expressions of IL-17RB, ST2 and TSLPR on CD1c^+^ DCs in NP tissues. Representative flow-cytometry pseudocolor density plots, in which gray-pink-blue indicates increasing cell density, showing the IL-17RB, ST2 and TSLPR expressions by CD1c^+^ DCs in nasal tissues from healthy controls (n = 16) and polyp tissues from NP patients (n = 30) (**a**). Quantification of IL-17RB, ST2 and TSLPR expressions by CD1c^+^ DCs (**b**). Data are presented as a percentage of the positive cells in CD1c^+^ DCs and mean with SD or median with IQR

The expressions of IL-17RB, ST2 and TSLPR on CD1c^+^ DCs were higher in NP tissues than in control tissues (Fig. 3a, b). Notably, the mean percentages of IL-17RB^+^ cells and ST2^+^ cells in the CD1c^+^ DCs in control tissues were 17.8% and 16.2% respectively, whereas in NP tissues were 77.6% and 64.5% respectively. By contrast, the mean percentage of TSLPR^+^ cells in the CD1c^+^ DCs in control tissues was 57.3%, whereas in NP tissues was 78.1%, suggesting CD1c^+^ DCs in nasal mucosa have a constitutional expression of TSLPR.

### Expression of OX40L, PDL1 and ICOSL on activated CD1c^+^ DCs in NP tissue

**Fig. 4:**
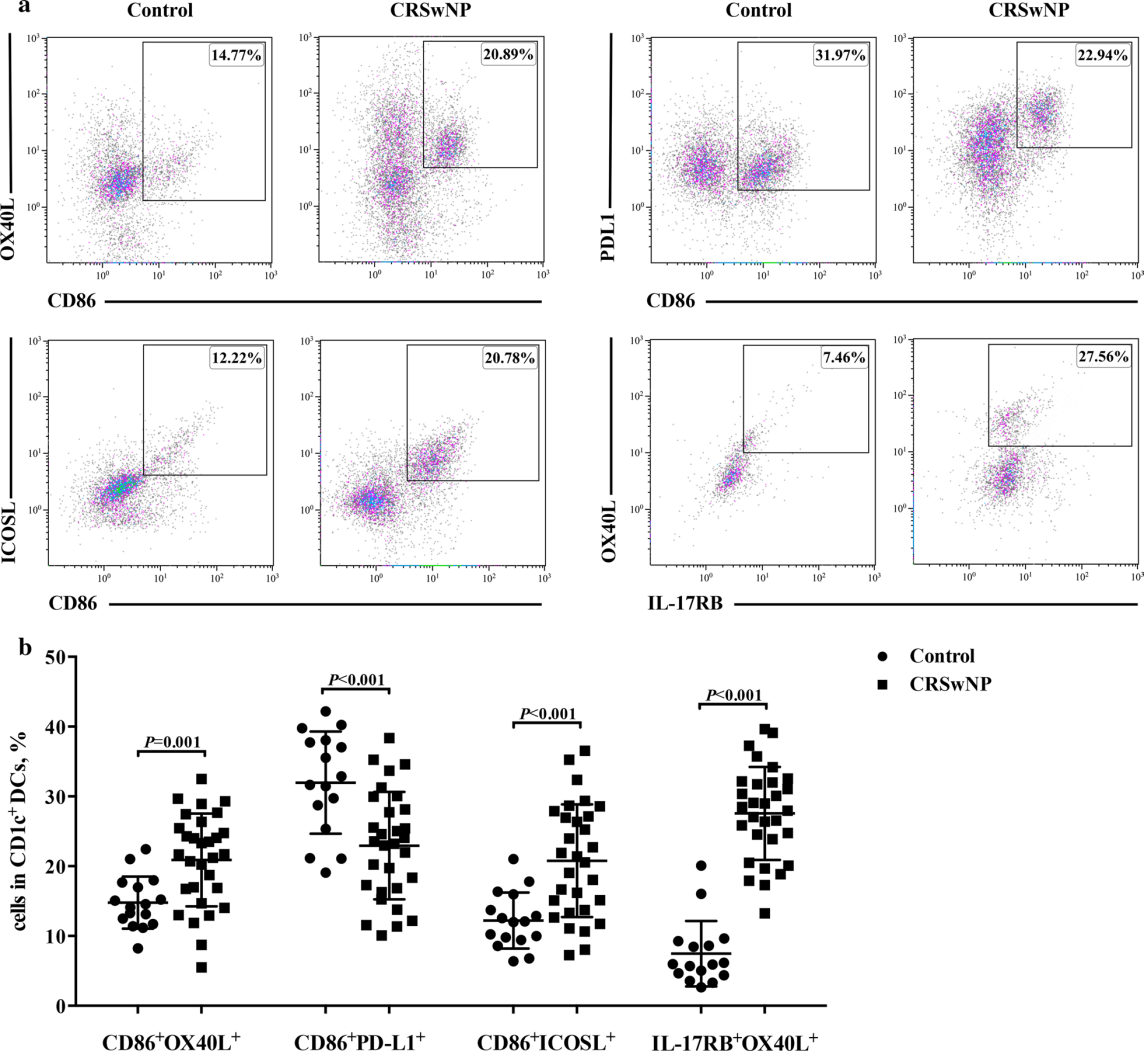
Expressions of OX40L, PDL1 and ICOSL on activated CD1c^+^ DCs in NP tissues. Representative flow-cytometry pseudocolor density plots, in which gray-pink-blue indicates increasing cell density, showing the percentages of CD86^+^OX40L^+^, CD86^+^PDL1^+^, CD86^+^ICOSL^+^ and IL-17RB^+^OX40L^+^ cells in CD1c^+^ DCs in nasal tissues from healthy controls (n = 16) and polyp tissues from NP patients (n = 30) (**a**). Quantification of CD86^+^OX40L^+^, CD86^+^PDL1^+^, CD86^+^ICOSL^+^ and IL-17RB^+^OX40L^+^ cells in CD1c^+^ DCs (**b**). Data are presented as a percentage of the positive cells in CD1c^+^ DCs and mean with SD or median with IQR

To further compare the phenotype of the activated CD1c^+^ DCs between NP and healthy control tissues, we analyzed the expression of OX40L, PDL1 and ICOSL on the CD86^+^CD1c^+^ DCs by flow cytometry. As expected, the expressions of OX40L and ICOSL were higher, but PDL1 was lower, on the CD86^+^CD1c^+^ DCs in NP tissues than their counterpart of control tissues (Fig. 4a, b). Moreover, OX40L expression on the IL-17RB^+^CD1c^+^ DCs was increased in NP tissues (Fig. 4a, b). However, no significant difference was found in OX40L expression on ST2^+^ CD1c^+^ or TSLPR^+^ CD1c^+^ DCs between NP and control tissues (data not shown).

### Correlations of functional phenotypes of CD1c^+^ DCs with cytokine mRNA expression profile in NP tissues

**Fig. 5:**
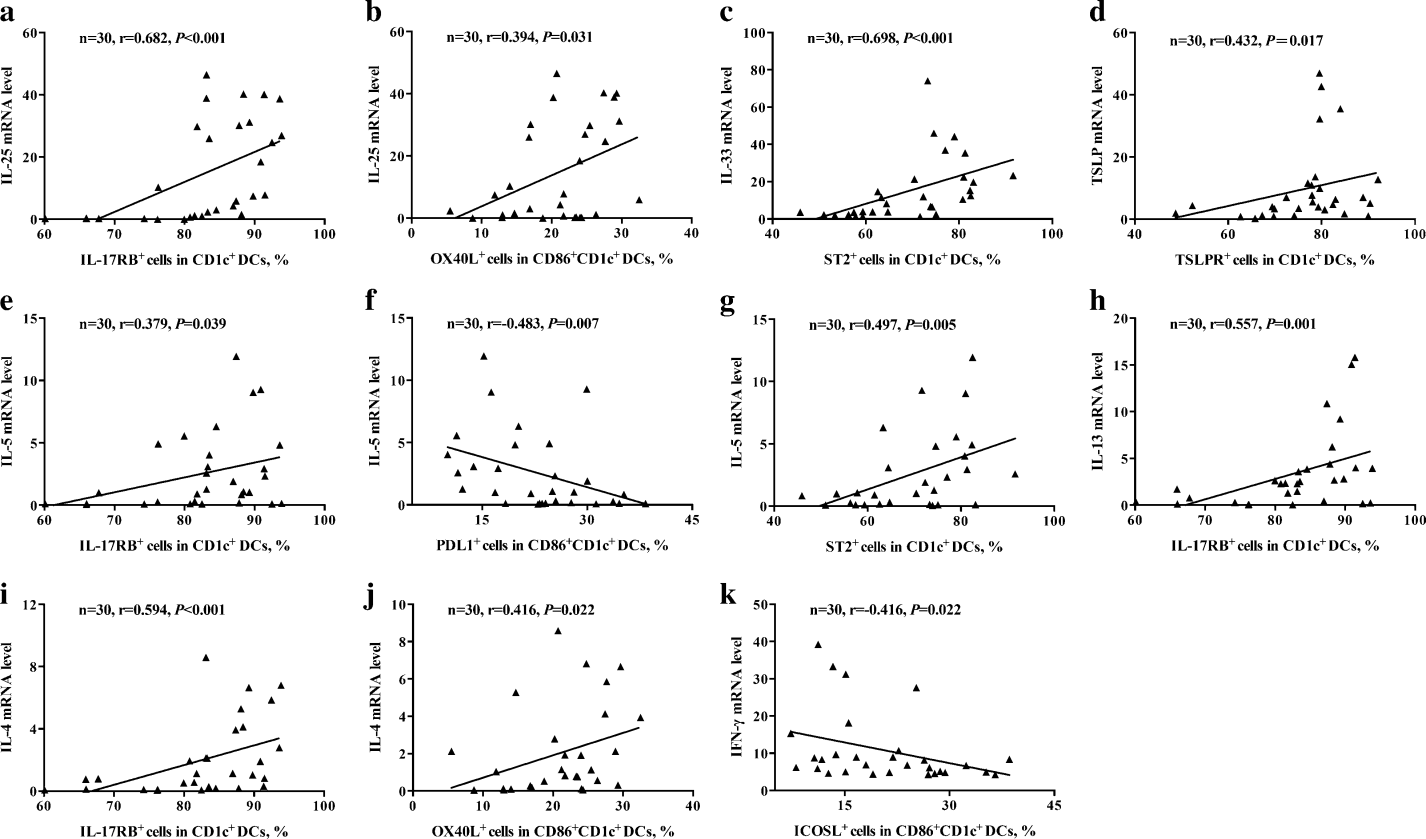
Correlation of DC surface phenotypes with cytokine mRNA expression levels in NP tissue. Correlations of the mRNA levels of IL-25 with the percentage of IL-17RB on CD1c^+^ DCs (**a**) and the percentage of OX40L on CD86^+^CD1c^+^ DCs (**b**), IL-33 with the percentage of ST2 on CD1c^+^ DCs (**c**), TSLP with the percentage of TSLPR on CD1c^+^ DCs (**d**), IL-5 with the percentage of IL-17RB on CD1c^+^ DCs (**e**), PDL1 on CD86^+^CD1c^+^ DCs (**f**) and ST2 on CD1c^+^ DCs (**g**), IL-13 with the percentage of IL-17RB on CD1c^+^ DCs (**h**), IL-4 with the percentage of IL-17RB on CD1c^+^ DCs (**i**) and OX40L on CD86^+^CD1c^+^ DCs (**j**), and IFN-γ with the percentage of ICOSL on CD86^+^CD1c^+^ DCs (**k**) in NP tissues

We next analyzed the relationship between IL-17RB, ST2 and TSLPR expressions, as well as other functional markers, on DC surface and TSLP, IL-25, IL-33 and Th2 cytokine mRNA expression levels in NP tissues. IL-17RB, ST2 and TSLPR expression on CD1c^+^ DCs were positively correlated with the mRNA expression of their ligands, IL-25, IL-33 and TSLP, respectively (r = 0.682, 0.698 and 0.432, respectively) (Fig. 5a, c, d). Importantly, IL-17RB expression on CD1c + DCs was positively correlated with Th2 cytokine mRNA levels in NP tissues, including IL-5, IL-13 and IL-4 (r = 0.379, 0.557 ad 0.594, respectively) (Fig. 5e, h, i). In addition, ST2 expression on CD1c^+^ DCs was positively correlated with IL-5 mRNA level in NP tissues (r = 0.497) (Fig. 5g). However, we did not find significant correlations between ST2 expression on CD1c^+^ DCs and IL-4 and IL-13 mRNA levels in NP tissues. Surprisingly, no significant correlations between TSLPR on CD1c^+^ DCs and Th2 cytokines were observed (Additional file 1: Table S3).

For the other functional markers on DC surface, we found that OX40L expression on CD86^+^CD1c^+^ DCs was positively correlated with IL-25 and IL-4 mRNA expression (r = 0.394 and 0.416) (Fig. 5b, j), but not with IL-33 or TSLP (Additional file 1: Table S3). Furthermore, PDL1 expression on CD86^+^CD1c^+^ DCs was negatively correlated with IL-5 mRNA level (r = − 0.483) (Fig. 5f). ICOSL expression on CD1c^+^ DCs was negatively correlated with IFN-γ mRNA level (r = − 0.416) (Fig. 5k).

### Correlations of DC surface phenotypes with disease severity in patients with NP

**Fig. 6:**
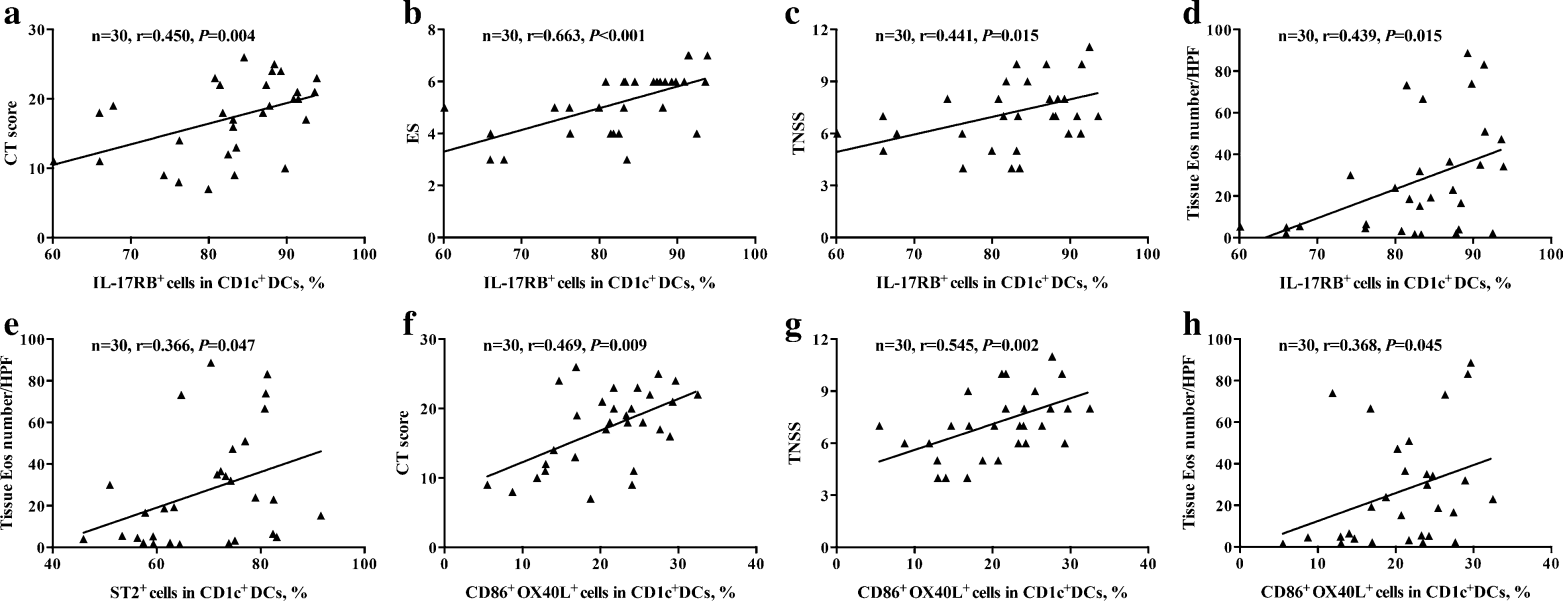
Correlations of DC surface phenotypes with disease severity in patients with NP. Correlations of the percentage of IL-17RB expression on CD1c^+^ DCs in NP tissues with CT scores (**a**), endoscopic scores (**b**), TNSS (**c**) and mean tissue eosinophil count (**d**). Correlation of the percentage of ST2 expression on CD1c^+^ DCs in NP tissues with mean tissue eosinophil count (**e**). Correlations of the frequencies of CD86^+^OX40L^+^ cells in CD1c^+^ DCs in NP tissues with CT scores (**f**), TNSS (**g**) and mean tissue eosinophil count (**h**). *CT* computed tomography, *ES* endoscopic score, *TNSS* total nasal symptom score, *Eos* eosinophil, *HPF* high-power field

Last, we analyzed the relationship between DC surface phenotypes and CT score, ES, TNSS and tissue eosinophil number. The correlation analyses are presented in Additional file 1: Table S4. IL-17RB expression on CD1c^+^ DCs was positively correlated with CT score, ES, TNSS and tissue eosinophil number, respectively (r = 0.450, 0.663, 0.441 and 0.439, respectively) (Fig. 6a–d). ST2 expression on CD1c^+^ DCs was positively correlated with tissue eosinophil number (r = 0.366) (Fig. 6e). In addition, OX40L expression on CD86^+^CD1c^+^ DCs was positively correlated with CT score, TNSS and tissue eosinophil number (r = 0.469, 0.545 and 0.368, respectively) (Fig. 6f–h).

## Discussion

The data presented in our study demonstrate that mDCs (CD1c^+^ DCs) accumulated in NP tissues expressed increased IL-17RB and ST2, which were positively correlated with their counterpart ligands IL-25 and IL-33 mRNA levels, as well as IL-5 mRNA level and eosinophil numbers in NP tissues. Given both IL-25 and IL-33 are predominantly produced by epithelial cells and have been shown to play an important role in the initiation and development of type 2 immune response [27], our data provide evidence for a link between mDCs and IL-25- and IL-33-induced type 2 responses and eosinophilic inflammation in NP.

NP has been known to be a Th2-skewed eosinophilic inflammation in the nasal sinus mucosa. T helper type 2-associated cytokines IL-4, IL-5 and IL-13 are involved in the pathogenesis of the eosinophilic airway diseases such as allergic asthma, allergic rhinitis and NP [37, 38]. IL-25, IL-33 and TSLP have recently been considered as potential therapeutic targets because of their important roles in initiating the type 2 inflammation [39]. In the present study, we found significant elevation in transcript levels of IL-25, IL-33 and TSLP, as well as IL-4, IL-5 and IL-13, but not IFN-γ and IL-17A, in patients with NP when compared with control subjects, which are largely consistent with previous reports [23, 25, 40], confirming the Th2-skewed innate and adaptive immune responses in the pathogenesis of NP.

DCs, particularly in the airway, play a critical role in the induction of peripheral tolerance and maintenance of immune homeostasis [41]. Therefore, DCs in the airway require some degree of activation to exert their function. This might explain why no difference in the percentage of activated mDCs in TMCs between patients with NP and control subjects was observed in the present study. In addition, we found PDL1 expression on CD86^+^CD1c^+^ DCs was lower in NP tissues than control tissues and negatively correlated with IL-5 mRNA level, this is in line with a recent study by Kortekaas et, al [42], showing that the PDL1 mRNA level was lower, but PD1 was higher and positively correlated with IL-5 mRNA level, in NP tissue. Furthermore, we found increased number of activated mDCs in PBMCs, but not in polyp tissues, in patients with NP, suggesting that the mDC pathogenicity might not be restricted to the local inflammatory responses in NP. However, these results are inconsistent with a recent study by Shi et, al, showing no significant difference in the percentage of mDCs and activated DC subsets between NP and control subjects [17]. The discrepancy may arise from differences in technical approaches. For example, we used CD1c as the marker of mDCs, whereas Shi et al. chose CD11c, which could also be expressed on some macrophage population [43].

From previous reports, IL-25, IL-33 and TSLP can affect the properties and functions of DCs. For example, IL-25 instructs DCs to promote Th2 and Th9 inflammation in mouse models of allergic airway inflammation [29, 44]. IL-33 activates DCs to express CCL17 and CCL22 through ST2 signaling [45]. TSLP-licensed DCs are responsible for the initiation of allergic airway inflammation [13, 28, 46]. However, whether DCs in NP respond to these innate type 2 cytokines remains unknown. In the present study, we demonstrated that mDCs accumulated in NP tissues exhibit elevated surface expression of IL-17RB, ST2 and TSLPR, suggesting that mDCs in NP have the potential to respond to IL-25, IL-33 and TSLP. These findings are parallel to our recent study in patients with allergic rhinitis [31].

Ample evidences have indicated that the signals provided by the surface of DC subsets dictate Th1-Th2 differentiation. For example, it has well been shown that surface expression of OX40L is critical for the induction and maintenance of type 2 immune response elicited by TSLP-activated DCs [13, 46]. PD-1/PDL1 interactions play an important role in maintaining peripheral tolerance [47, 48]. ICOSL has been reported to be involved in DC-driven Th2 response to allergens [49]. In our study, we found that activated mDCs expressed higher level of OX40L and ICOSL, but lower level of PDL1, in NP than those of control subjects. Furthermore, increased OX40L expression on activated mDCs was positively correlated with IL-4 and IL-25 mRNA levels, as well as tissue eosinophil numbers. In contrast, increased ICOSL and decreased PDL1 were negatively correlated with IFN-γ and IL-5 respectively. These results further suggest that mDCs might play a potential role in the IL-25- and IL-33-induced Th2 inflammation via expression of functional surface molecules, such as OX40L, ICOSL and PDL1, in NP.

Previous study showed that TSLP-induced OX40L expression on DCs is required for initiation of Th2 cell polarization, proposing an important role OX40L on DCs plays in determining T cell differentiation [46]. However, although increased expression of TSLP mRNA was noted in NP, we did not find significant association between TSLP mRNA level and OX40L expression on activated mDCs in NP, implying that TSLP might induce Th2 immune response not through OX40L expression on mDCs in NP. Further functional studies are needed to address this hypothesis.

Group 2 innate lymphoid cells (ILC2s) are a recently identified innate cell subset that produces large amounts of IL-5 and IL-13 and therefore serve an important role in orchestrating the type 2 inflammation [50–52]. IL-25, IL-33 and TSLP have been shown to be the key for production of type 2 cytokines by ILC2s [50–52]. Several studies have demonstrated that ILC2s are increased in NP tissues [24, 53, 54]. However, our recent study [26] found that the IL-17RB expression on ILC2 in NP was relatively low and unable to be upregulated by IL-25 in vitro, implying that ILC2 might not be critical in mediating IL-25-induced Th2 inflammation in NP. Whether this is the case for IL-33 remains to be investigated.

Although we have provided new information that elevated expression of IL-17RB and ST2 on mDCs might underlie the pathogenesis of IL-25- and IL-33-induced Th2 inflammation in NP, several limitations still need to be addressed before more definitive conclusions can be drawn. First, we were unable to perform functional experiments to analyze the direct effect of IL-25 and IL-33 on mDCs due to the technical limitations of the isolation of pure DCs from NP tissue. Second, the present study was an ex vivo study. It did not provide direct evidence of the role of IL-17RB and ST2 on mDCs in promoting Th2 inflammation in vivo. Third, the sample size is relatively small, since we did not observe several expected correlations such as between ST2 expression on mDCs and IL-4 and IL-13. Moreover, it should be noted that several correlations were weak, such as between OX40L expression on CD86^+^CD1c^+^ DCs and IL-25 mRNA level and tissue eosinophil number (r = 0.394 and 0.368, respectively), ST2 expression on CD1c^+^ DCs and tissue eosinophil number (r = 0.366), although the *P* values were less than 0.05. Further studies are needed to address these issues.

## Conclusion

In conclusion, myeloid DCs accumulated in NP showed a phenotype characterized by increased expression of IL-17RB, ST2 and TSLPR, which was positively correlated with the IL-25, IL-33 and TSLP mRNA levels in NP respectively. Furthermore, the IL-17RB and ST2 expressions on mDCs were also correlated with the IL-5 mRNA level and eosinophil numbers in NP tissues, suggesting that mDCs might play a role in IL-25- and IL-33-induced type 2 responses and eosinophilic inflammation in NP.

## Additional file

**Additional file 1: Table S1. ** Antibodies used for flow cytometry. **Table S2**. Primer sequences used for quantitative RT-PCR. **Table S3**. Correlations between DC surface phenotypes and cytokine mRNA expression levels in patients with NP (n = 30). **Table S4**. Correlations between DC surface phenotypes and disease severity in patients with NP (n = 30). **Table S5**. Total nasal symptom score.

## Abbreviations

CRS: chronic rhinosinusitis
CRSwNP: chronic rhinosinusitis with nasal polyps
CRSsNP: chronic rhinosinusitis without nasal polyps
CT: computerized tomography
DCs: dendritic cells
IL: interleukin
IQR: interquartile range
mDCs: myeloid dendritic cells
MHC: major histocompatibility complex
qPCR: qualitative polymerase chain reaction
SD: standard deviation
TSLPR: thymic stromal lymphopoietin receptor
Th: T helper
TMCs: tissue mononuclear cells
TNSS: total nasal symptom score
