# Supplementary material for: Elevated expression of IL-17RB and ST2 on myeloid dendritic cells is associated with a Th2-skewed eosinophilic inflammation in nasal polyps
Source: Clin Transl Allergy. 2018 Nov 29;8:50. doi: 10.1186/s13601-018-0237-4 (PMC6263180; doi:10.1186/s13601-018-0237-4)
Supplement: Supplementary file 1 — Additional file 1: Table S1. Antibodies used for flow cytometry. Table S2. Primer sequences used for quantitative RT-PCR. Table S3. Correlations between DC surface phenotypes and cytokine mRNA expression levels in patients with NP (n = 30). Table S4. Correlations between DC surface phenotypes and disease severity in patients with NP (n = 30). Table S5. Total nasal symptom score. [file 13601_2018_237_MOESM1_ESM.docx]

**Table S1. Antibodies used for flow cytometry**

| **Antibody** | **Fluorochrome** | **Manufacturer** | **Clone** | **Source** |
| --- | --- | --- | --- | --- |
| CD1c | PerCp-Cy5.5 | BD Biosciences | F10/21A3 | Monoclonal Mouse IgG1, k |
| CD86 | APC | BD Biosciences | FUN-1 | Monoclonal Mouse IgG1, k |
| IL-17RB | PE | R&D Systems | 170220 | Monoclonal Mouse IgG2b |
| TSLPR | Alexa Fluor 700 | R&D Systems | 147036 | Monoclonal Mouse IgG1 |
| OX40L | BV421 | BD Biosciences | ik-1 | Monoclonal Mouse IgG1, k |
| PDL1 | PE-Cy7 | BD Biosciences | MIH1 | Monoclonal Mouse IgG1, k |
| ICOSL | PE | BD Biosciences | 2D3/B7-H2 | Monoclonal Mouse IgG2b, k |
| ST2 | APC | R&D Systems | - | Polyclonal  Goat IgG |

**Table S2. Primer sequences used for quantitative RT-PCR**

|  | **Forward 5’-3’** | **Reverse 5’-3’** |
| --- | --- | --- |
| IL-25 | CCAGGTGGTTGCATTCTTGG | TGGCTGTAGGTGTGGGTTCC |
| IL-33 | AGCCTAGATGAGACACCGAATT | GGTCAGAAGGGATGGTAGGC |
| TSLP | CCCAGGCTATTCGGAAACTCA | ACGCCACAATCCTTGTAATTGTG |
| IFN-γ | TCGGTAACTGACTTGAATGTCCA | TCGCTTCCCTGTTTTAGCTGC |
| IL-4 | CACCGAGTTGACCGTAACAG | TCCTTCTCATGGTGGCTGTA |
| IL-5 | ATCTTTCAGGGAATAGGCACAC | CCGTCTTTCTTCTCCACACTTT |
| IL-13 | AGCATGGTATGGAGCATCAA | CAGCATCCTCTGGGTCTTCT |
| IL-17A | TCAACGCTGATGGGAACG | TCTTGCTGGATGGGGACA |
| GAPDH | TGCACCACCAACTGCTTAGC | GGCATGGACTGTGGTCATGAG |

**Table S3. Correlations between DC surface phenotypes and cytokine mRNA expression levels in patients with NP (n=30)**

|  | **r** | **P** |  | **r** | **P** |
| --- | --- | --- | --- | --- | --- |
| IL-25 mRNA and IL-17RB^+^ DCs | 0.682 | <0.001 | IFN-γ mRNA and TSLPR^+^ DCs | 0.126 | 0.507 |
| IL-25 mRNA and OX40L^+^ DCs | 0.394 | 0.031 | IFN-γ mRNA and OX40L^+^ DCs | -0.096 | 0.613 |
| IL-25 mRNA and PDL1^+^ DCs | -0.257 | 0.170 | IFN-γ mRNA and PDL1^+^ DCs | -0.287 | 0.124 |
| IL-25 mRNA and ICOSL^+^ DCs | -0.344 | 0.063 | IFN-γ mRNA and ICOSL^+^ DCs | -0.416 | 0.022 |
| IL-25 mRNA and IFN-γ mRNA | 0.114 | 0.551 | IL-4 mRNA and IL-17RB^+^ DCs | 0.594 | <0.001 |
| IL-25 mRNA and IL-4 mRNA | 0.562 | 0.001 | IL-4 mRNA and ST2^+^ DCs | 0.058 | 0.761 |
| IL-25 mRNA and IL-5 mRNA | 0.469 | 0.009 | IL-4 mRNA and TSLPR^+^ DCs | -0.265 | 0.164 |
| IL-25 mRNA and IL-13 mRNA | 0.525 | 0.003 | IL-4 mRNA and OX40L^+^ DCs | 0.416 | 0.022 |
| IL-25 mRNA and IL-17A mRNA | -0.396 | 0.062 | IL-4 mRNA and PDL1^+^ DCs | -0.425 | 0.019 |
| IL-33 mRNA and ST2^+^ DCs | 0.698 | <0.001 | IL-4 mRNA and ICOSL^+^ DCs | -0.163 | 0.389 |
| IL-33 mRNA and OX40L^+^ DCs | -0.120 | 0.526 | IL-5 mRNA and IL-17RB^+^ DCs | 0.379 | 0.039 |
| IL-33 mRNA and PDL1^+^ DCs | -0.287 | 0.124 | IL-5 mRNA and ST2^+^ DCs | 0.497 | 0.005 |
| IL-33 mRNA and ICOSL^+^ DCs | -0.388 | 0.064 | IL-5 mRNA and TSLPR^+^ DCs | -0.102 | 0.592 |
| IL-33 mRNA and IFN-γ mRNA | 0.249 | 0.184 | IL-5 mRNA and OX40L^+^ DCs | -0.101 | 0.595 |
| IL-33 mRNA and IL-4 mRNA | 0.140 | 0.459 | IL-5 mRNA and PDL1^+^ DCs | -0.483 | 0.007 |
| IL-33 mRNA and IL-5 mRNA | 0.389 | 0.034 | IL-5 mRNA and ICOSL^+^ DCs | 0.060 | 0.753 |
| IL-33 mRNA and IL-13 mRNA | 0.248 | 0.187 | IL-13 mRNA and L-17RB^+^ DCs | 0.557 | 0.001 |
| IL-33 mRNA and IL-17A mRNA | -0.196 | 0.299 | IL-13 mRNA and ST2^+^ DCs | 0.181 | 0.338 |
| TSLP mRNA and TSLPR^+^ DCs | -0.396 | 0.062 | IL-13 mRNA and TSLPR^+^ DCs | -0.224 | 0.234 |
| TSLP mRNA and OX40L^+^ DCs | 0.103 | 0.588 | IL-13 mRNA and OX40L^+^ DCs | 0.297 | 0.111 |
| TSLP mRNA and PDL1^+^ DCs | 0.098 | 0.608 | IL-13 mRNA and PDL1^+^ DCs | -0.165 | 0.383 |
| TSLP mRNA and ICOSL^+^ DCs | -0.075 | 0.692 | IL-13 mRNA and ICOSL^+^ DCs | -0.203 | 0.282 |
| TSLP mRNA and IFN-γ mRNA | 0.179 | 0.344 | IL-17A mRNA and L-17RB^+^ DCs | -0.309 | 0.097 |
| TSLP mRNA and IL-4 mRNA | -0.154 | 0.415 | IL-17A mRNA and ST2^+^ DCs | 0.003 | 0.986 |
| TSLP mRNA and IL-5 mRNA | -0.062 | 0.746 | IL-17A mRNA and TSLPR^+^ DCs | 0.149 | 0.055 |
| TSLP mRNA and IL-13 mRNA | 0.116 | 0.543 | IL-17A mRNA and OX40L^+^ DCs | 0.083 | 0.663 |
| TSLP mRNA and IL-17A mRNA | 0.103 | 0.590 | IL-17A mRNA and PDL1^+^ DCs | -0.161 | 0.395 |
| IFN-γ mRNA and IL-17RB^+^ DCs | -0.047 | 0.804 | IL-17A mRNA and ICOSL^+^ DCs | -0.002 | 0.994 |
| IFN-γ mRNA and ST2^+^ DCs | 0.378 | 0.097 |  |  |  |

The correlations were analyzed by Spearman’s rank correlation coefficient.

**Table S4. Correlations between DC surface phenotypes and disease severity in patients with NP (n=30)**

|  | **r** | **P** |  | **r** | **P** |
| --- | --- | --- | --- | --- | --- |
| CT score and IL-17RB^+^ DCs | 0.450 | 0.004 | TNSS and IL-17RB^+^ DCs | 0.441 | 0.015 |
| CT score and ST2^+^ DCs | -0.062 | 0.743 | TNSS and ST2^+^ DCs | -0.263 | 0.160 |
| CT score and TSLPR^+^ DCs | -0.053 | 0.780 | TNSS and TSLPR^+^ DCs | -0.207 | 0.273 |
| CT score and OX40L^+^ DCs | 0.469 | 0.009 | TNSS and OX40L^+^ DCs | 0.545 | 0.002 |
| CT score and PDL1^+^ DCs | 0.177 | 0.350 | TNSS and PDL1^+^ DCs | 0.293 | 0.116 |
| CT score and ICOSL^+^ DCs | -0.318 | 0.087 | TNSS and ICOSL^+^ DCs | -0.231 | 0.220 |
| ES and IL-17RB^+^ DCs | 0.663 | <0.001 | Tissue Eos and IL-17RB^+^ DCs | 0.439 | 0.015 |
| ES and ST2^+^ DCs | 0.201 | 0.288 | Tissue Eos and ST2^+^ DCs | 0.366 | 0.047 |
| ES and TSLPR^+^ DCs | -0.312 | 0.093 | Tissue Eos and TSLPR^+^ DCs | -0.060 | 0.752 |
| ES and OX40L^+^ DCs | 0.214 | 0.257 | Tissue Eos and OX40L^+^ DCs | 0.368 | 0.045 |
| ES and PDL1^+^ DCs | -0.036 | 0.852 | Tissue Eos and PDL1^+^ DCs | -0.161 | 0.396 |
| ES and ICOSL^+^ DCs | 0.008 | 0.968 | Tissue Eos and ICOSL^+^ DCs | -0.253 | 0.177 |

The correlations were analyzed by Spearman’s rank correlation coefficient. CT: computed tomography; ES: endoscopic score; TNSS: total nasal symptom score; Eos: eosinophil.

**Table S5.** Total nasal symptom score

| Symptoms | Scores |
| --- | --- |
| Nasal congestion | 0-3 |
| Loss of smell | 0-3 |
| Anterior rhinorrhea | 0-3 |
| Postnasal drip | 0-3 |
| TNSS | Out of 12 |

0 - Absent, 1 - Mild, 2 – Moderate, 3 – Severe, TNSS – Total nasal symptom score.
